# Alum as an Emetic

**Published:** 1854-01

**Authors:** 


Fom the N. H. Journal of Medicine.

*Alum as an Emetic.*

Dr. C. D. Meigs used Alum successfully to rid the stomach of opium taken the destroy life. An ounce of powdered opium had been swallowed; thirty grs. sulphate of zinc had been given without the slightest effect; two hours after "the patient was in a somnolent condition." "One ounce of powdered alum was immediately procured, and one half of it, mixed with a little syrup, was given to the patient, and followed in a few minutes by two or three tumblers of warm water, when copious vomiting ensued, by which a free discharge of the contents of the stomach was induced." The remedy was repeated, and the patient entirely recovered.

